# Extracting Teeth during Pregnancy

**Published:** 1871-08

**Authors:** H. E. Robinson


### ARTICLE III.

### *Extracting Teeth During Pregnancy.*

By Dr. H. E. Robinson.

It seem to be laid down a rule by nearly all surgical writers, and indeed is held in practice by nearly all surgeons and dentists, that teeth should not be extracted during pregnancy. Now we are all aware that the uterus is closely connected by the system with the teeth, and that this connection seems more sensitive when the uterus is in a gravid state. But it has always seemed to me that this fact was but another argument in favor of the extraction of the aching tooth, especially when the pain is caused by chronic periodontitis. I am not in favor of indiscriminate extraction of teeth, but will strive as hard as any dentist to save a tooth whenever possible. This should be done in all cases. But for periodontitis, I know of no surer cure than extraction. In the past two years I have had five patients who were pregnant, come to me aflicted with inflammation of the periosteum, some of whom had been kept awake for two nights, and in each case I extracted the offending tooth without any bad result whatever. In the last case, the woman was within about a month of her time for delivery, but she was suffering so acutely from the diseased tooth that I very carefully extracted it, and all was well. Therefore I conclude that where no other remedy is possible, extraction is better than prolonged suffering.

---

